# SOS teeth with advanced caries and sociodemographic indicators, health-related habits and dental attendance patterns: data from the Dental, Oral, Medical Epidemiological (DOME) nationwide records-based study

**DOI:** 10.1186/s12903-021-01751-5

**Published:** 2021-08-09

**Authors:** Itzhak Abramovitz, Avraham Zini, Ortal Kessler Baruch, Ron Kedem, Noam E. Protter, Boaz Shay, Nirit Yavnai, Dorit Zur, Eitan Mijiritsky, Galit Almoznino

**Affiliations:** 1 Faculty of Dental Medicine, Hebrew University of Jerusalem, Jerusalem, Israel; 2 Department of Endodontics, Hadassah Medical Center, Jerusalem, Israel; 3 Department of Community Dentistry, Hadassah Medical Center, Jerusalem, Israel; 4 Medical Information Department, General Surgeon Headquarter, Medical Corps, Israel Defense Forces, Tel-Hashomer, Israel; 5 Chief Dental Surgeon & Head of Forensic Unit, Medical Corps, Israel Defense Forces, Tel-Hashomer, Israel; 6 Medical Research & Academy Section, Medical Corps, Israel Defense Forces, Tel-Hashomer, Israel; 7 Department of Otolaryngology, Head and Neck and Maxillofacial Surgery, Tel-Aviv Sourasky Medical Center, Sackler Faculty of Medicine, Tel Aviv, Israel; 8 The Maurice and Gabriela Goldschleger School of Dental Medicine, Tel-Aviv University, Tel Aviv, Israel; 9 Head, Big Biomedical Data Research Laboratory, Faculty of Dental Medicine, Hebrew University of Jerusalem, P.O. Box 12272, 91120 Jerusalem, Israel; 10 Dean’s Office, Hadassah Medical Center, Jerusalem, Israel; 11 Department of Oral Medicine, Sedation & Maxillofacial Imaging, Hadassah Medical Center, Jerusalem, Israel

**Keywords:** Epidemiological study, Data mining, Electronic dental record, Caries, Carious lesion, SOS teeth, Socio-demographic, Socioeconomic, Dental attendance, Health-related habits

## Abstract

**Background:**

"SOS teeth" are teeth that need to be treated first, and represent dental teeth with deep caries seen clinically and radiographically which may require root canal treatment or extraction. The aims of the present research were to study the associations of SOS teeth with: socio-demographic parameters, dental attendance patterns, health-related habits among young to middle-aged adults.

**Methods:**

This cross-sectional records-based research analyzed data from the Dental, Oral, Medical Epidemiological (DOME) repository that captures comprehensive socio-demographic, medical, and dental databases of a nationwide sample of 132,529 records of dental attendees to military dental clinics for 1 year aged 18 to 50 years.

**Results:**

SOS teeth had a significant positive association in the multivariate analysis with male sex [OR 1.137, 95% Confidence Interval (CI): 1.079–1.199], rural versus urban Jewish locality [OR 1.748 (1.082–2.825)], and consumption of sweetened beverages [OR 1.415 (1.337–1.496)]. SOS teeth retained significant negative associations (protective parameter) with academic [OR 0.647 (0.592–0.708)] and technicians (OR 0.616 (0.556–0.682)] compared to high school education, high [OR 0.437 (0.401–0.476)], and medium (OR 0.648 (0.598–0.702)] versus low socio-economic status, urban non-Jewish versus urban Jewish locality [OR 0.746 (0.693–0.802)], Asia (OR 0.658 (0.452–0.959)], North America (OR 0.539 (0.442–0.658)] and Israel [OR 0.735 (0.686–0.788)] versus western Europe birth countries.

**Conclusions:**

Health authorities should be familiar with this profile of the patient who is vulnerable to SOS teeth and formulate policies and allow the appropriate implementation of strategies in those in high-risk populations.

## Background

Dental caries is a ubiquitous disease, affecting upwards of 35% of the global population, across all cultures, economic status, sex, and races [1]. Despite a decline in caries rates in certain European and American countries, the global burden of caries reported that in 2010, untreated caries in permanent teeth was the most prevalent condition worldwide, affecting 2.4 billion people [1]. In 2015, untreated caries in permanent dentition remained the most common health condition globally [2], making caries a health problem yet to be solved.

The Decayed Missing Filled (DMF) Surfaces/Teeth index is the most widely used tool in epidemiological studies [3]. However, it does not differentiate between the different stages of caries, making it impossible to plan and adopt effective strategies for disease control [4, 5]. In our recent publications, we coined the term "SOS teeth" to represent teeth that need to be treated first due to advanced caries reaching the pulp or the presence of decayed root fragments [5]. While the DMFT index does not contribute data on the clinical consequences of untreated dental caries, which include involvement of the pulp [6], the SOS teeth provide this information. Moreover, unlike the DMFT, assessment of SOS teeth includes inspection of dental radiographs, which is crucial for the identification of hidden caries [7]. Indeed, according to the protocol developed by Klein and colleagues [8] and modified by the World Health Organization [3], DMF is clinically examined in epidemiological studies using an explorer, mirror, and gauze, without X-ray imaging.

Our previous publications on SOS teeth described in detail the definition and concept of SOS teeth as well as their prevalence and distribution according to age and sex [5], and their association with the metabolic syndrome [9] among young to middle-aged adults. In the present study, we will further explore the association of SOS teeth with socio-demographic indicators, health-related behaviors, and dental attendance patterns. These comprehensive assessments are crucial since carious lesions are the result of the interconnection of numerous determinants and involve a dynamic process that occurs when the demineralization process predominates when the pathological factors outweigh the preventive factors [10]. Indeed, a recent meta-analysis of genome-wide association studies showed that the heritability of dental caries is enriched for conserved genomic regions and partially overlapping with a range of complex traits including smoking, education, personality traits, and metabolic measures [11]. It is now recognized that dental caries cannot be considered in isolation and its occurrence and control depend on the social environment and behavior, at the levels of the individual and the broader community [12]. It is therefore important to know the impact of social determinants on health, both at an individual and collective level, to plan actions at a local level [13]. In particular, it is important to assess the associations of socio-demographic, dental attendance patterns, and health-related habits concerning first priority teeth for treatment with advanced tooth caries, i.e. SOS teeth. To that end, the primary objective of the current research was to explore the associations between SOS teeth and socio-demographic characteristics, health-related behaviors, and dental attendance patterns among young to middle-aged adults from the military population representative of the general population in Israel. We hypothesized that SOS teeth morbidity is associated with lower socio-demographic status, worse health-related habits, and more no-shows to scheduled dental appointments.

Analyzing big data obtained from multiple patients is an evolving scientific field aimed to make breakthroughs in global medical research that will hopefully lead to evidence-based personalized medicine. An unusual opportunity exists in Israel to identify a typical profile of patients with advanced caries, using the extensive socio-demographic, dental medical information collected routinely in the military databases. Evaluating these would be beneficial for monitoring SOS teeth and giving the correct and immediate treatment in particular for those in high-risk populations by focusing interventions. Particularly in Israel, analyzing these data is an unmet need since in Israel there is no formal authority responsible for collecting data on dental and oral diseases [14]. This is despite the suggestion made by the Israeli ministry of health that there is a need for an epidemiological every 5 years [14]. Therefore, among the Israeli population, there is insufficient data concerning dental status and its trends throughout the years [14]. Therefore, analyzing the associations of SOS teeth with socio-demographic, dental attendance patterns and health-related habits, will provide essential data to health authorities needed to adopt specific strategies and allocate resources to target populations at high risk.

## Methods

The current study is a part of the Dental, Oral, Medical Epidemiological (DOME), a nation-wide cross-sectional records-based big data research [5, 9, 15]. The DOME comprehensive repository encompasses records of young to middle-aged adults aged 18 to 50 years in military service in the Israel Defense Forces (IDF) who attended the military dental clinics of the IDF for 1 year (2015) [5, 15]. In Israel, military service is obligatory for all eligible citizens over the age of 18 years. The medical and dental systems in the IDF are homogenous, with standardized uniform administrative as well as clinical process and treatment is free of charge [15]. All dental data is documented in the Dental Patient Record (DPR), the medical data is recorded in the CPR (a computerized patient record), and the sociodemographic is kept in the central demographic database [15]. In our previous publications, we detailed the data collection for the DOME repository [5, 9, 15]. Records were extracted simultaneously from these databases, and then the data was, prepared, cleaned, harmonized, anonymized, and transformed into an Excel file suitable for analyses. Subjects with available data in these systems were included in the study.

### Ethical approval

The Utilization of human subject data followed the requirements of the Medical Corps, Israel Defense Forces Institutional Review Board, and conforms to the STROBE guidelines (approval number: IDF-1281-2013) [5, 9, 15]. The need for informed consent was waived by the IRB considering that we retrospectively analyzed anonymous records.

### Study variables

#### The dependent variable

SOS teeth: was extracted from the DPR and defined as teeth that need to be treated first, with advanced caries that reach the pulp or the presence of residual root, as described in detail previously [5, 9]. This definition of SOS teeth corresponds to code number 6 of the Caries Assessment Spectrum and Treatment (CAST) tool for caries assessment [9, 16]. Nevertheless, dissimilar the original CAST, the diagnosis of SOS was with the aid of compressed air and included radiographs [9, 16, 17].

#### Independent variables

Independent variables included were defined as follows:

*The criteria for the social-demographic variables* Socio-demographic data were drawn from the central socio-demographic database of the IDF. Definitions of the sociodemographic variables included in the DOME repository are provided elsewhere [15]. The following variables as follows: (1) Education: high school/technician/academic, (2) Socio-economic status (SES): low, medium, high [18], (3) birth country: Western Europe, East Europe, and the former Soviet Union (FSU), Asia, Ethiopia, Africa, North America, South America, and Israel. (4) Rings of a city/town (midtown/suburbs), (5) Locality of residence: urban Jewish/ urban non-Jewish/rural.

*Definitions of dental attendance* These variables evaluate dental health care utilization during the past year, and were extracted from the DPR [15]: The total number of attended dental appointments, and the total number of non-attendance to scheduled dental appointments.

*Definitions of self-reported health-related habits* (1) Extracted from the DPR (yes/no): brushing teeth once a day or more, cariogenic diet and sweetened beverages consumption, as described previously [15], (2) Extracted from the CPR: Current smoking and alcohol consumption status (yes/no).

### Methods of statistical analysis

The IBM SPSS® software version 25.0 (Chicago, Illinois, United States) was used to perform statistical analyses. Means and standard deviations were used to present numerical variables. Frequencies and percentages were used to describe categorical variables.

Statistical analyses of SOS teeth with the independent variables consisted of the following: Independent t-test or Analysis of variance (ANOVA) and Post Hoc Bonferroni tests for categorial parameters and Pearson's correlation for continuous parameters. Post Hoc Bonferroni tests were used in all parameters with more than two categories (Tables 2 and 3) to address the probability of at least one Type I error in the set of comparisons.

### Analytical statistics- addressing the large sample size and possible confounders

Following the univariate analyses, a multivariate analysis was performed using linear regression analysis for SOS teeth as the dependent variable, with independent variables. The criteria for independent variables to enter the multivariate analysis were:

(1) A statistically significant association with SOS teeth in the univariate analysis [*p* < 0.01(2-tailed) due to the large sample size]. (2) Lack of collinearity of the independent variables. Multicollinearity tests between the variables comprising the DOME repository have been previously described [15].

Finally, the multivariate linear regression analysis was used as a statistical model, by including in the analysis simultaneously all the variables fulfilling the criteria to enter the analysis as described above. Due to the large sample size, a *p* value of < 0.01 (2-tailed) was also considered statistically significant in the multivariate analysis.

## Results

This records-based research included 132,529 dental attendees. The prevalence of subjects with SOS teeth was 9.16% (12,146 out of 132,529). As described previously, the number of teeth that were found to be SOS teeth was 18,300. Therefore, the mean number of SOS teeth per a diseased patient (i.e., a patient with at least one SOS tooth) was 1.5 (18,300/12,146), and the mean number of SOS teeth in the whole study population was 0.14 ± 0.52 [5].

### The associations of SOS teeth with socio-demographic characteristics

SOS teeth were analyzed according to socio-demographic parameters using independent t-test and ANOVA analysis (Table 1) and post hoc Bonferroni tests (Tables 2 and 3) for categorial parameters, and Pearson's correlation for continuous parameters.

**Table 1:** The association of SOS teeth with socio-demographic parameters

Parameter	Variable	N	Mean	Std. deviation	95% Confidence interval for mean		*p* value
					Lower bound	Upper bound	
Sex	Female	33,063	0.12	0.46	0.11	0.12	< 0.001*
	Male	99,466	0.15	0.54	0.14	0.15	
	Total	132,529	0.14	0.52	0.14	0.14	
Education	High school	112,112	0.15	0.54	0.14	0.15	**< 0.001^**
	Technicians	7426	0.10	0.39	0.09	0.11	
	Academic	12,816	0.08	0.35	0.08	0.09	
	Total	132,354	0.14	0.52	0.14	0.14	
Socio-economic status (SES)	Low	5719	0.27	0.76	0.25	0.29	**< 0.001^**
	Medium	68,619	0.16	0.55	0.15	0.16	
	High	56,707	0.10	0.44	0.10	0.11	
	Total	131,045	0.14	0.52	0.14	0.14	
Birth country	Western Europe	10,571	0.19	0.61	0.18	0.20	**< 0.001^**
	East Europe and FSU	1715	0.22	0.65	0.18	0.25	
	Asia	509	0.11	0.44	0.07	0.15	
	Ethiopia	2185	0.19	0.63	0.17	0.22	
	Africa	345	0.14	0.54	0.08	0.20	
	North America	2859	0.08	0.37	0.06	0.09	
	South America	957	0.14	0.61	0.10	0.18	
	Israel	113,359	0.13	0.51	0.13	0.14	
	Total	132,500	0.14	0.52	0.14	0.14	
Rings of a city/town	Peripheral (suburbs)	118,450	0.14	0.52	0.14	0.14	< 0.001*
	Central (midtown)	14,079	0.12	0.48	0.11	0.13	
	Total	132,529	0.14	0.52	0.14	0.14	
Locality of residence	Urban Jewish	113,468	0.14	0.53	0.14	0.15	**< 0.001^**
	Urban non-Jewish	17,918	0.10	0.43	0.09	0.11	
	Rural	583	0.11	0.38	0.07	0.14	
	Total	131,969	0.14	0.52	0.14	0.14	

Parameter	Mean ± SD	Range	Pearson correlation (R)	*p* value**
Age (years)	21.5 ± 5.5	18–50	− 0.033	< 0.001

*Independent t-test; ^ANOVA; **Pearson's correlation; SD: standard deviation

**Table 2:** Post Hoc Bonferroni tests of the mean number of SOS teeth according to socio-demographic parameters

Parameter	Variable	Variable	Mean difference	Std. error	*p* value	95% Confidence Interval	
						Lower bound	Upper bound
Education	High school	Technicians	0.046	0.006	**< 0.001**	0.03	0.06
		Academic	0.065	0.005	**< 0.001**	0.05	0.08
	Technicians	Academic	0.019	0.008	0.051	0.00	0.04
Socio-economic status (SES)	Low	Medium	0.116	0.007	**< 0.001**	0.10	0.13
		High	0.170	0.007	**< 0.001**	0.15	0.19
	Medium	High	0.054	0.003	**< 0.001**	0.05	0.06
Locality of residence	Urban Jewish	Urban non-Jewish	0.044	0.004	**< 0.001**	0.03	0.05
		Rural	0.038	0.022	0.217	− 0.02	0.09
	Rural	Urban non-Jewish	0.006	0.022	0.961	− 0.05	0.06

Bold values represent statisticaly significant p values

**Table 3:** Post Hoc Bonferroni tests of the mean number of SOS teeth according to birth countries

Parameter	Variable	Mean difference	Std. error	*p* value	95% Confidence interval of mean	
					Lower bound	Upper bound
Western Europe	East Europe and FSU	− 0.027	0.014	0.797	− 0.08	0.02
	Asia	0.079	0.024	0.132	− 0.01	0.17
	Ethiopia	− 0.003	0.012	1.000	− 0.05	0.04
	Africa	0.047	0.029	0.910	− 0.06	0.15
	North America	0.111	0.011	**< 0.001**	0.07	0.15
	South America	0.050	0.018	0.324	− 0.02	0.12
	Israel	0.056	0.005	**< 0.001**	0.04	0.08
East Europe and FSU	Asia	0.106^*^	0.026	**0.024**	0.01	0.20
	Ethiopia	0.024	0.017	0.958	− 0.04	0.09
	Africa	0.074	0.031	0.572	− 0.04	0.19
	North America	0.138	0.016	**< 0.001**	0.08	0.20
	South America	0.077	0.021	0.065	0.00	0.16
	Israel	0.083	0.013	**< 0.001**	0.04	0.13
Asia	Ethiopia	− 0.082	0.026	0.181	− 0.18	0.01
	Africa	− 0.032	0.036	0.998	− 0.17	0.10
	North America	0.032	0.025	0.976	− 0.06	0.13
	South America	− 0.029	0.029	0.994	− 0.14	0.08
	Israel	− 0.023	0.023	0.995	− 0.11	0.06
Ethiopia	Africa	0.050	0.030	0.911	− 0.06	0.16
	North America	0.114	0.015	**< 0.001**	0.06	0.17
	South America	0.053	0.020	0.449	− 0.02	0.13
	Israel	0.059	0.011	**< 0.001**	0.02	0.10
Africa	North America	0.064	0.030	0.698	− 0.05	0.18
	South America	0.003	0.033	1.000	− 0.12	0.13
	Israel	0.009	0.028	1.000	− 0.10	0.11
North America	South America	− 0.061	0.019	0.194	− 0.13	0.01
	Israel	− 0.055	0.010	**< 0.001**	− 0.09	− 0.02
South America	Israel	0.006	0.017	1.000	− 0.06	0.07

Bold values represent statisticaly significant p values

*SOS teeth were negatively associated with*: age, with a weak correlation coefficient (Pearson Correlation = − 0.033, *p* < 0.001) (Table 1)

SOS teeth were positively associated with:

1. Male sex (*p* < 0.001) (Table 1).
2. Education (Tables 1 and 2):
  - High school versus technician education (*p* < 0.001).
  - High school versus academic education (*p* < 0.001). A dose–response trend was shown for the education parameter, with a higher mean number of SOS associated with lower education levels (Tables 1 and 2).
3. SES (Tables 1 and 2):
  iii. Low versus medium SES (*p* < 0.001)
  iv. Low versus high SES (*p* < 0.001).
  v. Medium versus high SES (*p* < 0.001).
    A dose–response trend was shown for the SES parameter, with a higher mean number of SOS associated with lower SES levels.
4. Birth countries (Tables 1 and 3):
  - Western Europe versus North America (*p* < 0.001) and Israel (*p* < 0.001).
  - East Europe and FSU versus Asia (*p* = 0.024), North America (*p* < 0.001), and Israel (*p* < 0.001).
  - Ethiopia versus North America (*p* < 0.001) and versus Israel (*p* < 0.001).
  - Israel versus North America (*p* < 0.001).
5. Living in the peripheral (suburbs) versus central (midtown) of a city/town (*p* < 0.001) (Table 1).
6. The locality of residence (Tables 1 and 2): urban Jewish versus urban non-Jewish (*p* < 0.001).

### The associations of SOS teeth with health-related habits

The statistically significant associations of SOS teeth with health-related habits are presented in Table 4. Brushing teeth at least once a day was negatively associated (protective effect) with the mean number of SOS teeth (*p* < 0.001). SOS teeth were positively associated with the consumption of a cariogenic diet (*p* < 0.001) and sweetened beverages (*p* < 0.001) (Table 4).

**Table 4:** Associations of SOS teeth with health-related habits

Domain	Parameter	Variable	N	Mean	SD	95% confidence interval for mean		*p* value*
						Lower bound	Upper bound	
Health-related habits	Brushing teeth once a day	No	5357	0.22	0.66	0.20	0.24	**< 0.001**
		Yes	39,676	0.13	0.49	0.13	0.14	
		Total	45,033	0.14	0.52	0.14	0.15	
	Cariogenic diet	No	22,003	0.13	0.48	0.12	0.14	**< 0.001**
		Yes	22,975	0.16	0.55	0.15	0.17	
		Total	44,978	0.14	0.52	0.14	0.15	
	Sweetened beverages	No	20,432	0.11	0.44	0.11	0.12	**< 0.001**
		Yes	24,487	0.17	0.57	0.16	0.18	
		Total	44,919	0.15	0.52	0.14	0.15	

	Parameter	Pearson correlation ( R)	*p* value^
Dental attendance patterns	Number of dental visits	0.109	**< 0.001**
	Number of non-attendances to scheduled dental appointments	0.080	**< 0.001**

^*^Independent t-test; ^Pearson's correlation; SD: standard deviation; Bold values represent statistically significant *p* values

Smoking (*p* = 0.858) and alcohol consumption (*p* = 0.878) had no statistically significant associations with SOS teeth.

### The associations of SOS teeth with dental attendance patterns

The statistically significant associations of SOS teeth with dental attendance patterns are presented in Table 4. SOS teeth had a significant positive correlation with: the number of dental visits (Pearson Correlation = 0.109; *p* < 0.001) and with the number of non-attendances to scheduled dental appointments (Pearson Correlation = 0.080; *p* < 0.001) (Table 4). The Pearson Correlation's coefficient of these analyses are considered negligible.

In summary, the results of the univariate analyses identified the following profile of patients with higher mean number of SOS teeth: male sex, younger age, lower education (high school vs. technician and academic education), lower SES (low vs. medium and high SES, and medium vs. high) urban Jewish versus urban non-Jewish locality, birth countries from Western Europe, East Europe and FSU Ethiopia, living in the peripheral versus central of a city/town, brushing teeth less than once a day, consumption of cariogenic diet and sweetened beverages.

### Multivariate analysis of the mean number of SOS teeth with statistically significant parameters

**Fig. 1:**
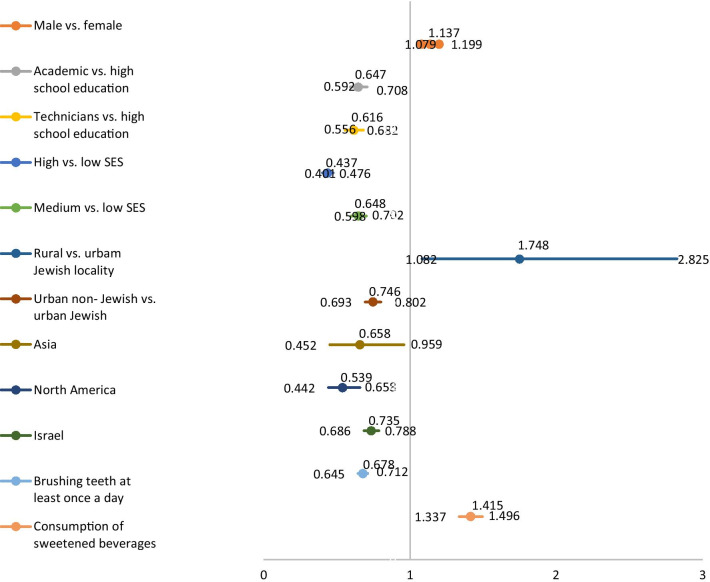
Forest plot presenting the multivariate linear regression analysis of the mean number of SOS teeth with statistically significant parameters

Parameters fulfilling the criteria to enter the multivariate analysis (as described in the statistical section), were included in a multivariate linear regression model. The parameters that retained a statistically significant association with SOS teeth in the multivariate analysis are presented in the forest plot in Fig. 1.

Parameters that retained a statistically significant positive association with SOS teeth in the multivariate analysis (Fig. 1):

- Male versus female sex [*p* < 0.001, Odds Ratio (OR) 95% & Confidence Interval: 1.137 (1.079 to 1.199)]
- Rural versus urban Jewish locality [*p* = 0.0226, OR & 95% Confidence Interval: 1.748 (1.082 to 2.825)]
- Consumption of sweetened beverages [*p* =  < 0.001, OR & 95% Confidence Interval: 1.415 (1.337 to 1.496)].

Parameters that retained a significant negative association (protective variable) with SOS teeth in the multivariate analysis (Fig. 1):

1. Education:
  - Academic versus high school education [*p* < 0.001, OR & 95% Confidence Interval: 0.647 (0.592 to 0.708)].
  - Technicians versus high school education [*p* < 0.001, OR & 95% Confidence Interval: 0.616 (0.556 to 0.682)].
2. SES
  - High versus low SES [*p* < 0.001, OR & 95% Confidence Interval: 0.437 (0.401to 0.476)]
  - Medium versus low SES [*p* < 0.001, OR & 95% Confidence Interval: 0.648 (0.598 to 0.702)].
3. Locality
  Urban non-Jewish versus urban Jewish [p<0.001, OR & 95% Confidence Interval: 0.746 (0.693 to 0.802)].
4. Birth country
  Compared Western Europe birth countries:
  - Asia [*p* = 0.0293, OR & 95% Confidence Interval: 0.658 (0.452 to 0.959)].
  - North America [*p* < 0.001, OR & 95% Confidence Interval: 0.539 (0.442 to 0.658)]
  - Israel [*p* < 0.001, OR & 95% Confidence Interval: 0.735 (0.686 to 0.788)].
5. Brushing teeth at least once a day [p(<0.001, OR & 95% Confidence Interval: 0.747 (0.645 to 0.712)] (Table 4).

Parameters that lost the association with SOS teeth in the multivariate analysis (Fig. 1):

*Cariogenic diet consumption* (*p* = 0.1576) lost its statistical significance association with SOS teeth in the multivariate analysis.

In summary, the forest plot in Fig. 1 demonstrates the results of the multivariate analysis. Risk factors for higher mean number of SOS teeth were male sex, rural versus urban Jewish locality and consumption of sweetened beverages. Protective factors were academic and technician education, high and medium SES, urban non- Jewish locality, brushing teeth at least once a day and birth countries from Asia, North America, and Israel.

## Discussion

SOS teeth are teeth with advanced carious lesions that require more complicated treatments that may include filling, treatment of the root canals, or extraction and are defined as the first priority for treatment. In our previous publications, we coined the term "SOS teeth", and analyzed its associations with age and sex differences [5] and with metabolic syndrome [9]. In the present study, we furthermore analyzed the associations of SOS teeth with socio-demographic characteristics, dental attendance patterns, and health-related habits. These comprehensive assessments of factors associated with SOS teeth are important since the discriminating factors leading and linked to different stages of dental caries are still unclear [4]. The epidemiology of dental diseases has clearly been described as a web of connecting factors, including biological, social, psychological, economic, environmental, and other variables [19]. Thanks to the multivariable analysis, that addressed multiple possible confounders, we identified a typical profile of patients with “SOS teeth” among a nation-wide population of young to middle-aged adults that includes: male sex, lower education, lower SES, Jewish urban and rural localities, brushing teeth less than once a day, and consumption of sweetened beverages. Protective factors were birth countries from Asia, North America, and Israel. To the best of our knowledge, this is the first study in English literature to identify a comprehensive patient profile among a large sample of patients with advanced caries in Israel. The profile of a patient that is more likely to have SOS teeth that will be discussed below.

### Associations of socio-demographic parameters with SOS teeth

#### Age and sex

In our previous publication, we explored in detail the age and sex differences in the prevalence of SOS teeth in this population [5]. In the present study, we further explored more socio-demographic indicators and considered the age and sex parameters as important co-variants in the analysis. While SOS teeth were negatively associated with age in a statistically significant manner (*p* < 0.001), the correlation coefficient was negligible (Pearson Correlation coefficient = − 0.033), and therefore age was not included in the multivariate analysis. Sex was included in the multivariate analysis and retained its statistical significance with SOS teeth even following multivariate analysis. Sex differences in the prevalence of SOS teeth could be attributed to biological factors present during reproductive age, as well as to higher rates of trauma to dentition among males in general and in particular among the military population [5]. Nevertheless, many risk factors for caries may induce a sex bias towards a higher caries risk among women compared to men [20]. Among these factors are hormonal fluctuations, differences in nutritional practices, and the composition of saliva and in salivary flow, social roles among the family, and genetic variations [21]. It should be noted that sex differences have narrowed over the past 20 years due to changes in society and culture, amelioration of education among females, and enhanced focus on women's health [22].

#### Education

In the current study, lower education was associated with SOS teeth. Per our results, Celeste et al. found a greater probability of non-treated caries lesion in low educated people and attributed their results to the fact that educated people exhibited better knowledge of caries [23]. Higher education is associated with higher general and oral health literacy [24]. Studies demonstrated that individuals with low health literacy include the poor and those with low levels of education [25]. A previous study conducted among a representative sample of 7139 21-year-old Israeli adults between 1994 and 1997, found that caries scores were higher among subjects with less than 12 years of schooling [26].

#### Socio-economic status (SES)

In the present study, subjects with SOS teeth were more likely to be in the low SES clusters. In line with our findings, others also demonstrated that there is an impact of earlier lower SES on later caries disease [27]. Indeed, previous studies have shown that those in the lower-income brackets are likely to be at a higher risk for caries and tooth loss [28]. A persistently low SES was associated with the greatest risk of both new and accumulated caries [29]. Moreover, a recent systematic review and meta-regression demonstrated a positive association between low SES and severe caries [30]. The reasons for the association of low SES with caries could be attributed to the impact of SES on three major determinants: health behaviors, environmental exposures and health care [30]. Social disadvantage is associated with health inequalities, barriers of access to oral health care and poor literacy in oral health which lead to a sustained vulnerability due to deficient support for good oral health habits in exposed [29, 30]. Boillot et al. concluded that identifying socioeconomic at-risk populations for oral diseases enhances the effectiveness of preventive campaigns by focusing interventions, adopting specific strategies, and obtaining active participation of target populations [31].

#### Locality

The current study demonstrated that subjects with SOS teeth are more likely to be residents of rural versus urban, and within urban in Jewish more than in non-Jewish localities. Supporting our results, a recent WHO survey demonstrated that carious lesions are left without treatment mostly in rural areas [32]. Perinetti et al. demonstrated that caries prevalence in the primary and the permanent dentition of school children living in rural areas is significantly higher than that of children living in urban areas, suggesting that in older subjects the urbanization level may become a strong indicator for dental caries [33].

#### Birth country

Israel is known as an immigrant country, with the largest increase of immigrants in the nineties from the former Soviet Union (FSU) and Ethiopia. Our findings found significantly fewer SOS teeth in subjects from Asia, North America, and Israel compared to Western Europe. The highest mean number of SOS teeth was found in subjects from East Europe and FSU, followed by Ethiopia and Western Europe. In line with our results, a study conducted among a representative sample of 21-year-old Israeli adults found higher levels of caries experience among soldiers of Ethiopian or FSU origin compared to native Israelis [26]. Another Israeli study assessed caries levels among 581 young adults during their military service and showed that caries experience was higher among immigrants from the FSU, compared to native Israelis [34]. Also, a study conducted by dental records of army recruits between 2012 and 2013, found a statistically significant association between higher treatment needs among subjects whose parents came from Ethiopia or FSU [35]. Supporting our findings of higher prevalence of SOS teeth in those originated from western Europe, Edelstein et al. found that globally, the caries map showed a clear pattern of higher disease experience in western Europe, but they also found in the north and south America, and much of Africa [28].

### Health-related habits and SOS teeth

#### Sweetened beverages and cariogenic diet

This study analyzed the exposure to sugars not only in foods but also to beverages and found a statistically significant association between SOS teeth and consumption of sweetened beverages and cariogenic diet. Interestingly, sweetened beverages, but not a cariogenic diet, retained a statistical significance with SOS teeth in the multivariable analysis. The assertion that diet plays a central role in the development of dental caries is unquestionable [36]. The frequent and high consumption of sugar products, particularly sucrose, is one of the well-known causative factors of dental caries [37]. In agreement with our findings, the Vipeholm dental caries study [38] showed that sugar increased caries most if consumed between meals, and in a form that was retained for a long time in the mouth. Moreover, Ismail et al. found a significant positive association between the frequencies of at- and between-meal consumption of soft drinks and high DMFT scores [39]. Furthermore, children aged 2 to 10 years with a predominantly high soft drink diet, were found to be 1.8 times more likely to experience dental caries in the primary dentition [40]. However, a review and a meta-analysis that analyzed the association between soft drink consumption and caries demonstrated only a negligible positive (*r* = 0.03) [41]. A possible explanation could be exposure to fluoridated public water, which helped ameliorate the association between sugar-sweetened beverages consumption and dental decay [42]. In Israel, fluoride was required in water supplies nationwide by legislation passed in 2002 [43], and this practice continues, except for a short period between 2014 and 2015 when the requirement was repealed, and resumed 1 year later, in 2015. Indeed, the present study demonstrated that native Israelis had a relatively lower prevalence of SOS teeth, which can be credited, at least in part, to water fluoridation. Being an immigrants country means that many current citizens were not necessarily exposed to fluoridated water in their birth country.

#### Brushing teeth once a day

Our study demonstrated that teeth brushing at least once a day was negatively associated with the number of SOS teeth (protective factor). Tooth-brushing twice a day has become a social norm, but the evidence base for this frequency is weak [44]. Our findings correspond with studies showing that self-reported infrequent brushers demonstrate higher incidence and increment of carious lesions than frequent brushers [44]. Coparal et al. showed that the percentage of children who never or irregularly brushed their teeth was highest in the caries active group [45]. After adjusting for other confounders in the multivariate analysis (Fig. 1), tooth brushing retained a statistically significant association with SOS teeth, emphasizing its fundamental role as a self-care behavior for maintaining oral health.

#### Tobacco smoking

Smoking had no statistically significant association with SOS teeth. Abundant research focused on tobacco smoking effects on oral health. On one hand, there are reports that following an increase in smoking the caries rate decreased [46]. However, other studies reported that those who smoked had a statistically significant higher DMFT [47]. Multiple other factors can contribute directly or indirectly to dental caries prevalence in smokers such as age, oral hygiene habits, eating habits, drinking habits, visits to the dentist, which makes it is difficult to establish the strength of the relationship between smoking and dental caries [48].

#### Alcohol consumption

In this study, the association between alcohol consumption and SOS teeth was not statistically significant. A lower rate of alcohol heavy consumers (i.e. alcoholism) among the military population could account for this observation.

#### Dental attendance and SOS teeth

There was a weak positive correlation between SOS teeth and the number of dental appointments, as well as with non-attendance to scheduled dental appointments. However, due to the weak correlations, dental attendance parameters were not included in the multivariate analysis. Similar to our findings, others also reported that higher DMFT was found among irregular dental attendees, and both SES and dental attendance were independently affecting the DMFT [49]. The WHO has observed that developed countries have higher rates of caries experience while developing countries have lower rates and attributed these differences to the relative availability of simple sugars in diets, fluoride, and dental treatments [23].

#### Strengths and limitations

The main strengths of this study are the large sample size (132,529 dental records and 12,146 subjects with SOS teeth) including the meticulous protocol for utilization of the extensive databases. Definitions of socio-demographics are strict and uniform for all IDF personnel. For assessment of SOS teeth, both clinical and radiographic examinations were included. Moreover, the records of SOS teeth were based on dental examinations that were according to uniform guidelines of the IDF medical corps dental division applied on all subjects, and all military dentists went through uniform training. Socio-demographic parameters, dental attendance patterns, and dental and oral morbidities were records-based, eliminating the recall bias associated with self- reported data. However, health-related habits were recorded in the databases according to the patients' reports and are therefore subjected to recall bias. The cross-sectional study design means that causality cannot be assumed, and consequently, we present only associations and correlations between the variables. Although dental assessment followed a uniform protocol, an optimal calibration was not attainable and there could be possible variations in the diagnoses and determination of priorities for treatment of carious lesions. Moreover, despite the accessibility of dental examinations in the IDF, being free of charge for all military personal and obligatory for are combatant during military training in the first four months, there are cases where patients miss the examinations or choose to be treated in a civilian dental clinic, which can lead to under documentation, since we could only measure SOS teeth among dental attendees.

## Conclusions

This extensive nationally representative study aimed to identify a typical profile of patients with “SOS teeth” in terms of socio-demographic characteristics, health-related behaviors, and dental attendance patterns among young to middle-aged adults. Following the multivariable analysis, we identified a patient profile predictive of the SOS teeth which include: male sex, lower education, lower SES, Jewish urban and rural localities, brushing teeth less than once a day, and consumption of sweetened beverages. Protective factors were birth countries from Asia, North America, and Israel.

To conclude, the dentist and health authorities should be familiar with the profile of the patient who is vulnerable to SOS teeth. Assessment and treatment should consider all the risk factors for SOS teeth and adjust the treatment approach in the fields of general and dental health-related risk factors. Identifying socioeconomic at-risk populations for oral diseases can enhance health authorities and health care providers' ability to formulate policies and implement interventions and raise new objectives for research which is crucial to establish evidence-based guidelines.

## Data Availability

The data that support the findings of this study are available from the Medical Corps, Israel Defense Forces, Israel, but restrictions apply to the availability of these data, which were used under license for the current study, and so are not publicly available. Data are however available from the authors upon reasonable request and with permission of the Medical Corps, Israel Defense Forces.

## Abbreviations

CPR: Clinical Patient Record
DMFT: Decayed, missed, filled teeth
DPR: Dental Patient Record
FSU: Former Soviet Union
SES: Socio-economic status

## References

[1] Kassebaum NJ, Bernabe E, Dahiya M, Bhandari B, Murray CJ, Marcenes W (2015). Global burden of untreated caries: a systematic review and metaregression. J Dent Res.

[2] Peres MA, Macpherson LMD, Weyant RJ, Daly B, Venturelli R, Mathur MR, Listl S, Celeste RK, Guarnizo-Herreno CC, Kearns C (2019). Oral diseases: a global public health challenge. Lancet.

[3] World Health Organization: Oral health surveys: basic methods. 5th ed. https://www.who.int/publications/i/item/9789241548649.

[4] Campus G, Cocco F, Strohmenger L, Cagetti MG (2020). Caries severity and socioeconomic inequalities in a nationwide setting: data from the Italian National pathfinder in 12-years children. Sci Rep.

[5] Almoznino G, Abramovitz I, Kessler Baruch O, Kedem R, Protter NE, Levine J, Bader T, Yavnai N, Zur D, Mijiritsky E (2020). SOS teeth: age and sex differences in the prevalence of first priority teeth among a national representative sample of young and middle-aged adults. Int J Environ Res Public Health.

[6] Monse B, Heinrich-Weltzien R, Benzian H, Holmgren C, van Palenstein HW (2010). PUFA–an index of clinical consequences of untreated dental caries. Community Dent Oral Epidemiol.

[7] Zadik Y, Bechor R (2008). Hidden occlusal caries: challenge for the dentist. N Y State Dent J.

[8] Klein H, Palmer C, Knutson J (1938). Studies on dental caries: I. Dental status and dental needs of elementary school children. Public Health Rep (1896–1970).

[9] Almoznino G, Kessler Baruch O, Kedem R, Protter NE, Shay B, Yavnai N, Zur D, Mijiritsky E, Abramovitz I (2020). SOS teeth: first priority teeth with advanced caries and its associations with metabolic syndrome among a national representative sample of young and middle-aged adults. J Clin Med.

[10] Cappelli DP, Mobley CC (2008). Prevention in clinical oral health care.

[11] Shungin D, Haworth S, Divaris K, Agler CS, Kamatani Y, Keun Lee M, Grinde K, Hindy G, Alaraudanjoki V, Pesonen P (2019). Genome-wide analysis of dental caries and periodontitis combining clinical and self-reported data. Nat Commun.

[12] Rugg-Gunn A (2013). Dental caries: strategies to control this preventable disease. Acta Med Acad.

[13] Vazquez Fde L, Cortellazzi KL, Kaieda AK, Bulgareli JV, Mialhe FL, Ambrosano GM, da Silva Tagliaferro EP, Guerra LM, de Castro MM, Pereira AC (2015). Individual and contextual factors related to dental caries in underprivileged Brazilian adolescents. BMC Oral Health.

[14] Ministry of Health Israel. Oral and dental health- goals for year 2020 (In Hebrew). https://www.health.gov.il/PublicationsFiles/2020_18052016.pdf.

[15] Almoznino G, Kedem R, Turgeman R, Bader T, Yavnai N, Zur D, Shay B (2020). The Dental, Oral, Medical Epidemiological (DOME) study: protocol and study methods. Methods Inf Med.

[16] Frencken JE, de Amorim RG, Faber J, Leal SC (2011). The Caries Assessment Spectrum and Treatment (CAST) index: rational and development. Int Dent J.

[17] Frencken JE, de Souza AL, van der Sanden WJ, Bronkhorst EM, Leal SC (2013). The caries assessment and treatment (CAST) instrument. Community Dent Oral Epidemiol.

[18] Peled A, Gordon B, Twig G, Grossman E, Matani D, Derazne E, Afek A. Hypertension and childhood migration: a nationwide study of 2.7 million adolescents. J Hypertens. 2018;37:702–9. 10.1097/HJH.0000000000001957 30817450

[19] Marmot M, Bell R (2011). Social determinants and dental health. Adv Dent Res.

[20] Lukacs JR (2011). Sex differences in dental caries experience: clinical evidence, complex etiology. Clin Oral Investig.

[21] Lukacs JR, Largaespada LL (2006). Explaining sex differences in dental caries prevalence: saliva, hormones, and "life-history" etiologies. Am J Hum Biol.

[22] Kassebaum NJ, Bernabe E, Dahiya M, Bhandari B, Murray CJ, Marcenes W (2014). Global burden of severe tooth loss: a systematic review and meta-analysis. J Dent Res.

[23] Celeste RK, Nadanovsky P, De Leon AP (2007). Association between preventive care provided in public dental services and caries prevalence. Rev Saude Publica.

[24] Batista MJ, Lawrence HP, Sousa MDLR. Oral health literacy and oral health outcomes in an adult population in Brazil. BMC Public Health. 2017;26;18(1):60. 10.1186/s12889-017-4443-0 PMC5530456 28747157

[25] Horowitz AM, Kleinman DV. Oral health literacy: the new imperative to better oral health. Dent Clin North Am. 2008;52(2):333–44, vi. 10.1016/j.cden.2007.12.001 18329447

[26] Sgan-Cohen HD, Katz J, Horev T, Dinte A, Eldad A (2000). Trends in caries and associated variables among young Israeli adults over 5 decades. Community Dent Oral Epidemiol.

[27] Nicolau B, Marcenes W, Bartley M, Sheiham A (2003). A life course approach to assessing causes of dental caries experience: the relationship between biological, behavioural, socio-economic and psychological conditions and caries in adolescents. Caries Res.

[28] Edelstein BL. The dental caries pandemic and disparities problem. BMC Oral Health. 2006;6 Suppl 1(Suppl 1):S2. 10.1186/1472-6831-6-S1-S2. 10.1186/1472-6831-6-S1-S2 PMC2147591 16934119

[29] Ostberg AL, Petzold M (2020). A longitudinal study of the impact of change in socioeconomic status on dental caries in the permanent dentition of Swedish children and adolescents. Community Dent Oral Epidemiol.

[30] Costa SM, Martins CC, Pinto MQC, Vasconcelos M, Abreu M (2018). Socioeconomic factors and caries in people between 19 and 60 years of age: an update of a systematic review and meta-analysis of observational studies. Int J Environ Res Public Health.

[31] Boillot A, El Halabi B, Batty GD, Range H, Czernichow S, Bouchard P (2011). Education as a predictor of chronic periodontitis: a systematic review with meta-analysis population-based studies. PLoS ONE.

[32] Ondine Lucaciu P, Mester A, Constantin I, Orban N, Cosma L, Candrea S, Sava-Rosianu R, Mesaros AS (2020). A WHO pathfinder survey of dental caries in 6 and 12-year old transylvanian children and the possible correlation with their family background, oral-health behavior, and the intake of sweets. Int J Environ Res Public Health.

[33] Perinetti G, Varvara G, Esposito P (2006). Prevalence of dental caries in schoolchildren living in rural and urban areas: results from the first region-wide Italian survey. Oral Health Prev Dent.

[34] Birnboim-Blau G, Levin L, Sgan-Cohen HD: Dental status among native and immigrant young Israeli adults. Refuat Hapeh Vehashinayim (1993). 2006;23(1):6–11, 67. 16599327

[35] Levy DH, Livny A, Sgan-Cohen H, Yavnai N (2018). The association between caries related treatment needs and socio-demographic variables among young Israeli adults: a record based cross sectional study. Israel J Health Policy Res.

[36] Burt BA, Pai S. Sugar consumption and caries risk: a systematic review. J Dent Educ. 2001;65(10):1017–23. 11699972

[37] Olczak-Kowalczyk D, Turska A, Gozdowski D, Kaczmarek U (2016). Dental caries level and sugar consumption in 12-year-old children from Poland. Adv Clin Exp Med.

[38] Gustafsson BE, Quensel CE, Lanke LS, Lundqvist C, Grahnen H, Bonow BE, Krasse B (1954). The Vipeholm dental caries study; the effect of different levels of carbohydrate intake on caries activity in 436 individuals observed for five years. Acta Odontol Scand.

[39] Ismail AI, Burt BA, Eklund SA (1984). The cariogenicity of soft drinks in the United States. J Am Dent Assoc.

[40] Sohn W, Burt BA, Sowers MR (2006). Carbonated soft drinks and dental caries in the primary dentition. J Dent Res.

[41] Vartanian LR, Schwartz MB, Brownell KD (2007). Effects of soft drink consumption on nutrition and health: a systematic review and meta-analysis. Am J Public Health.

[42] Armfield JM, Spencer AJ, Roberts-Thomson KF, Plastow K (2013). Water fluoridation and the association of sugar-sweetened beverage consumption and dental caries in Australian Children. Am J Public Health.

[43] Zusman SP (2014). Water fluoridation in Israel: ethical and legal aspects. Public Health Rev.

[44] Kumar S, Tadakamadla J, Johnson NW (2016). Effect of toothbrushing frequency on incidence and increment of dental caries: a systematic review and meta-analysis. J Dent Res.

[45] Eronat N, Koparal E. Dental caries prevalence, dietary habits, tooth-brushing, and mother's education in 500 urban Turkish children. J Marmara Univ Dent Fac. 1997;2(4):599–604. 9569784

[46] Schmidt HJ (1951). Tobacco smoke and the teeth. Stoma (Heidelb).

[47] Ludwick W, Massler M (1952). Relation of dental caries experience and gingivitis to cigarette smoking in males 17 to 21 years old (at the Great Lakes Naval Training Center). J Dent Res.

[48] Vellappally S, Fiala Z, Smejkalova J, Jacob V, Shriharsha P (2007). Influence of tobacco use in dental caries development. Cent Eur J Public Health.

[49] Tickle M, Williams M, Jenner T, Blinkhorn A (1999). The effects of socioeconomic status and dental attendance on dental caries' experience, and treatment patterns in 5-year-old children. Br Dent J.

